# Radiological outcomes in perianal fistulizing Crohn's disease: A systematic review and meta‐analysis

**DOI:** 10.1002/jgh3.12295

**Published:** 2019-12-30

**Authors:** Tanya Lee, Eric Yong, Nik S Ding

**Affiliations:** ^1^ St Vincent's Clinical School University of Melbourne Melbourne Victoria Australia; ^2^ Department of Radiology St Vincent's Hospital Melbourne Victoria Australia; ^3^ Department of Gastroenterology St Vincent's Hospital Melbourne Victoria Australia

**Keywords:** biological therapy, Crohn's disease, magnetic resonance imaging, rectal fistula, tumor necrosis factor‐alpha

## Abstract

Perianal fistulas are a common and debilitating manifestation of Crohn's disease. Since the advent of biological agents, patient outcomes appear to have improved. While rates of clinical response and remission are well characterized in literature, magnetic resonance imaging (MRI) outcomes remain less so. This is despite previous studies demonstrating the persistence of fistula tracts on MRI, in spite of clinical healing, suggesting radiological markers of improvement may be more accurate. The aims of this study were to systematically review the literature for all studies reporting on MRI outcomes following biological therapy and to compare rates of radiological healing to clinical remission. A search was performed according to the Preferred Reporting Items For Systematic Reviews and Meta‐Analysis (PRISMA) guidelines. Nine articles were included, with a total sample size of 259 patients. Of these 259 patients, 47% achieved clinical remission following induction therapy and 42% following a median of 52 weeks' maintenance therapy. Out of the 259 patients, 7% achieved radiological healing in the short term and 25% in the long term. The odds ratio of MRI *versus* clinical healing was 0.10 (95% confidence interval [CI], 0.02–0.39) and 0.43 (95% CI, 0.26–0.71), respectively, at those corresponding time points. MRI healing of perianal fistulizing Crohn's, while arguably a more accurate assessment of treatment response, is significantly less common than clinical remission. Heterogeneity exists in the definition of radiological and clinical response, leading to variation in reported rates. Further studies, directly comparing the long‐term outcomes of patients achieving clinical remission and MRI healing are required, to better inform the role of MRI follow up in clinical practice.

## Graphical abstract

Perianal fistulizing Crohn's remains a significant clinical challenge, with a high burden of disease and relative treatment resistance. Magnetic resonance imaging outcomes, while more accurate and objective, remain uncharacterised. This systematic review with meta‐analysis presents the current evidence regarding the radiological response to anti‐TNF alpha treatment.

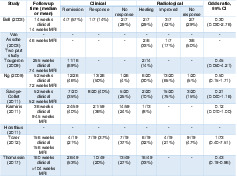

## Introduction

Perianal fistulizing Crohn's disease (pfCD) is a common and debilitating phenotype,^1^ with its relative treatment resistance rendering it a significant clinical challenge. Treatment is optimized with combined medical and surgical intervention.^2^ Prior to the advent of biological agents, medical therapy consisted of antibiotics and nonbiological immunomodulators (thiopurines), leading to a proctectomy rate of 40%.^3^ Since the introduction of biological therapy, particularly the anti‐TNFα agents, patient outcomes appear to have improved, leading to better quality of life^1^ and decreased hospitalizations.^4^

Two broad categories of outcomes have been used in the literature to assess the success of pfCD treatment, the first being clinical findings based on the closure of fistula external openings, established by Present *et al*.'s landmark randomized controlled trial.^5^ Clinical remission rates of 55% following induction therapy^5^ and 36% following 1 year of maintenance therapy^6^ are commonly cited.

The second group of outcomes assessed are radiological, based on magnetic resonance imaging (MRI) appearance on T2‐weighted sequences. Composite scores, such as the van Assche score, have been proposed, incorporating anatomical (number of tracts, location, presence/absence of levatoric extension) and inflammatory features (degree of T2 hyperintensity, presence/absence of collections, rectal wall involvement) as a marker of disease severity.^7^ Underlying fistula tracts may persist despite clinical healing, suggesting that MRI may be a more accurate measure of disease activity.^7^, ^8^ Despite this, the radiological outcomes of pfCD remain uncharacterized, and the role of MRI in driving clinical decision‐making is unclear.

The aim of this study was to systematically review current literature for all studies reporting on MRI outcomes following anti‐TNFα therapy and to compare rates of radiological healing with clinical remission.

## Methods

This review was conducted according to the Preferred Reporting Items for Systematic Reviews and Meta‐Analyses (PRISMA) guidelines.^9^

### *Search strategy*

The online databases Medline (1946 to February 2018), EMBASE (1947 to February 2018), and Cochrane Library were searched. The following Medical Subject Headings (MeSH) and keywords were used, alone or in combination: Crohn's disease, anal fistula, perianal fistula, rectal fistula, biological therapy, tumour necrosis factor, adalimumab, infliximab, drug therapy, surgical therapy. Results were limited to English language, and the adult population. Abstracts of all potentially relevant publications were consulted, and full texts of all eligible articles were obtained. Additional studies were obtained from searching cited references of selected articles.

### *Selection criteria*

All publications assessing MRI outcomes in perianal fistulas secondary to Crohn's disease treated with biological therapy, alone or with adjunctive therapy, were included in the review. Study designs were limited to randomized controlled trials (RCTs), cohort studies, and case–control studies.

### *Data analysis*

In studies assessing pfCD alongside other fistulizing subtypes of Crohn's disease, only patients with perianal subtypes were included in the study population. Assessments (clinical and/or radiological) occurring ≤12 weeks after treatment commencement were considered short term and those >12 weeks long term. In studies where multiple assessments occurred within the short‐ or long‐term period, the results of the assessment with the longest follow‐up period were entered into analysis. Results of patients on infliximab and adalimumab were analyzed together, given previous studies demonstrating that, when used as first‐line therapy, these agents are equally efficacious.^10^ Data were entered into RevMAN version 5.3 (Copenhagen, The Nordic Cochrane Centre, The Cochrane Collaboration, 2014), and an odds ratio of clinical *versus* radiological healing in the short and long term was calculated.

## Results

**Figure 1:**
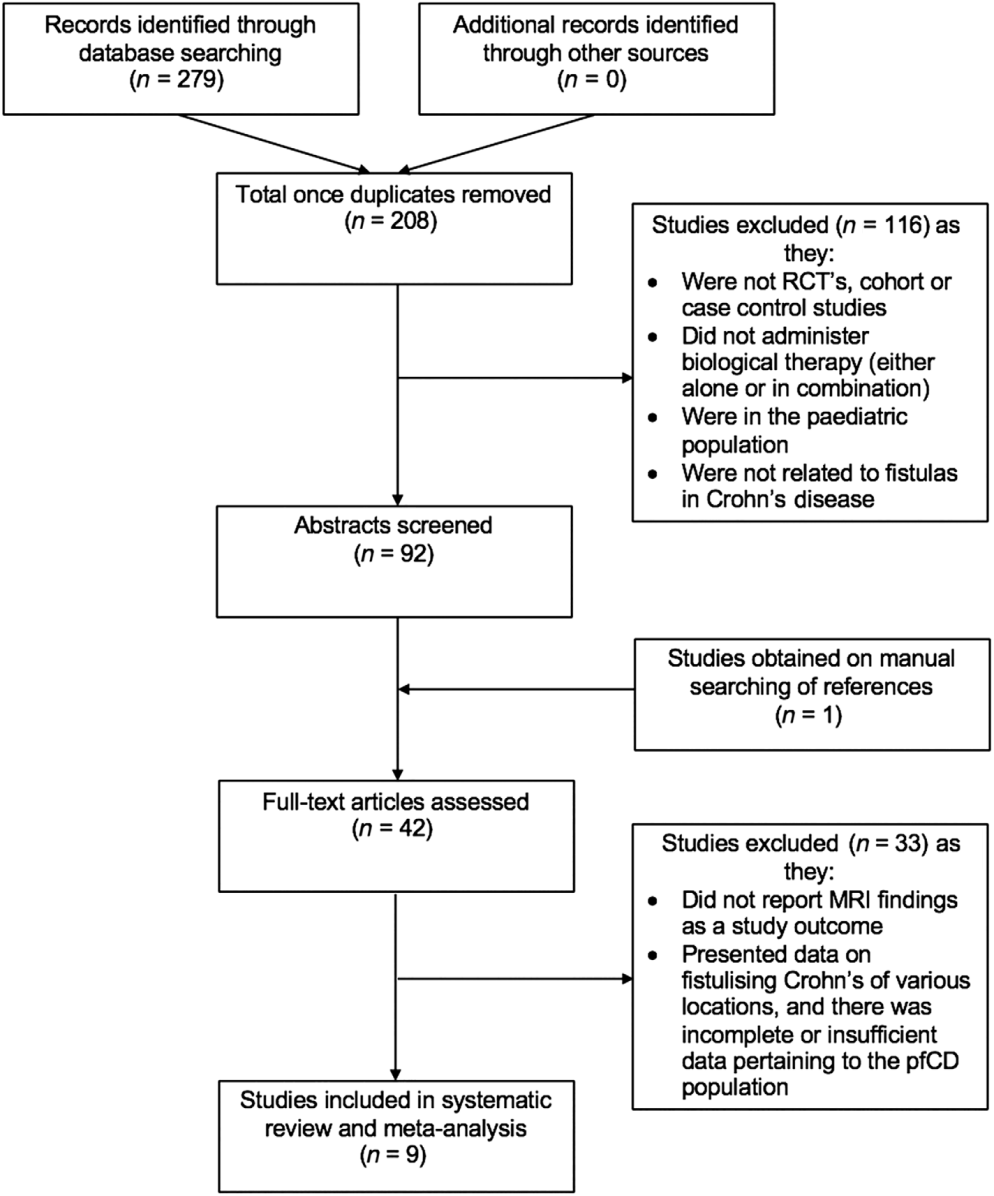
A PRISMA diagram outlining the search and study selection methods.^9^

Electronic search returned an initial total of 208 papers with duplicates removed; no studies were obtained via other sources. A total of 116 studies were excluded on title review as they were not RCTs/cohort/case–control studies, did not administer biological therapy (either alone or in combination), were in the pediatric population, or were not related to fistulas in Crohn's disease. A total of 92 abstracts were screened, and a further 84 publications were excluded as MRI findings were not a study outcome, or they presented data on fistulizing Crohn's of various locations (of which pfCD was a subtype), and there was incomplete/insufficient data pertaining to the pfCD population. An additional article was obtained from the citation lists. Nine studies were included in the systematic review (see Fig. 1).

Across the nine studies, a total of 259 pfCD patients were treated (see Table 1). Of patients, 43% were male and 57% female, with a median/mean age of 34 years. A total of 184 patients received induction therapy with infliximab and 26 with adalimumab; 179 patients received maintenance therapy with infliximab and 39 with adalimumab.

**Table 1:** Patient demographics and biological agents used in the studies

Study and year of publication	Number of pfCD patients	Gender	Mean/median age (years)	Induction therapy	Maintenance therapy
Bell *et al*.^8^	7	3 males/4 females	39	7 infliximab	—
Van Assche *et al*.^7^ (two part study)	8	—	—	8 infliximab	—
	7	—	—	7 infliximab	7 infliximab
Tougeron *et al*.^16^	26	—	—	26 infliximab	16 infliximab
Ng *et al*.^1^, ^11^	26	10 males/16 females	—	19 infliximab/7 adalimumab	19 infliximab/7 adalimumab
Savoye‐Collet *et al*.^17^	20	6 males/14 females	34	10 infliximab/10 adalimumab	10 infliximab/10 adalimumab
Karmiris *et al*.^12^	59	22 males/37 females	23	59 infliximab	59 infliximab
Horsthius *et al*.^13^	16	9 males/7 females	34	16 infliximab	—
Tozer *et al*.^14^	41	16 males/25 females	36	32 infliximab/9 adalimumab	25 infliximab/16 adalimumab
Thomassin *et al*.^15^	49	28 males/21 females	33	43 infliximab/6 adalimumab	43 infliximab/6 adalimumab

pfCD, perianal fistulising Crohn's disease.

### *Clinical outcomes*

Of the nine studies, all examined clinical outcomes, with heterogeneity in end‐points assessed (see Tables 2 and 3). A fistula was defined as “closed” in the absence of drainage, with gentle finger compression on examination. Nine papers defined “remission” as closure of all baseline fistulas; six studies required this over two consecutive evaluations^8^, ^11^, ^12^, ^13^, ^14^, ^15^ and three at a single time point.^7^, ^16^, ^17^ Eight studies assessed for clinical “response.” This was defined as closure of ≥50% of baseline fistulas at a single time point in three papers,^7^, ^15^, ^17^ and across two consecutive visits in three papers.^8^, ^12^, ^13^ In two papers, it was defined as either closure of ≥50% baseline draining fistulas **or** marked reduction in drainage of all fistulas with less pain and induration, as reported by the patient, across two consecutive visits.^11^, ^14^

**Table 2:** Short‐term (≤12 weeks) clinical and radiological outcomes

		Clinical, *n* (%)			Radiological, *n* (%)			
Study	Follow‐up time (median or mean)	Remission	Response	No response	Healing	Improved	No response	Odds ratio, 95% CI
Bell *et al*.^8^	—	—	—	—	—	—	—	—
Van Assche *et al*.^7^ Two part study	6 weeks clinical 6 weeks MRI	4/8 (50%)	1/8 (13%)	3/8 (38%)	0/8 (0%)	2/8 (25%)	6/8 (75%)	0.030 (0.00–0.56)
	10 weeks clinical 10 weeks MRI	4/7 (57%)	2/7 (29%)	1/7 (14%)	0/7 (0%)	6/7 (86%)	1/7 (14%)	—
Tougeron *et al*.^16^	8–12 weeks clinical	13/26 (50%)	—	—	—	—	—	—
Ng *et al*.^1^, ^11^	—	—	—	—	—	—	—	—
Savoye‐Collet *et al*.^17^	—	—	—	—	—	—	—	—
Karmiris *et al*.^12^	11 weeks MRI	—	—	—	3/29 (10%)	—	—	—
Horsthius *et al*. (2011)	12 weeks clinical 12 weeks MRI	6/16 (38%)	1/16 (6%)	9/16 (56%)	1/16 (6%)	—	—	0.17 (0.030–0.90)
Tozer *et al*.^14^	—	—	—	—	—	—	—	—
Thomassin *et al*.^15^	—	—	—	—	—	—	—	—

CI, confidence interval; MRI, magnetic resonance imaging.

**Table 3:** Long‐term (>12 weeks) clinical and radiological outcomes

		Clinical, *n* (%)			Radiological, *n* (%)			
Study	Follow up time (median or mean)	Remission	Response	No response	Healing	Improved	No response	Odds ratio, 95 CI
Bell *et al*.^8^	14 weeks clinical 14 weeks MRI	4/7 (57%)	1/7 (14%)	2/7 (29%)	2/7 (29%)	3/7 (42%)	2/7 (29%)	0.30 (0.030–2.76)
Van Assche *et al*.^7^ Two part study	—	—	—	—	—	—	—	—
	46 weeks MRI	—	—	—	2/6 (33%)	1/7 (17)	3/6 (50%)	—
Tougeron *et al*.^16^	255 weeks clinical 74 weeks MRI	11/16 (69%)	—	—	2/14 (14%)	—	—	0.45 (0.050–4.21)
Ng *et al*.^1^, ^11^	52 weeks clinical 72 weeks MRI	12/26 (46%)	13/26 (50%)	1/26 (4%)	6/20 (30%)	13/20 (65%)	1/20 (5%)	0.50 (0.15–1.71)
Savoye‐Collet *et al*.^17^	52 weeks clinical 52 weeks MRI	7/20 (35%)	8/20 (40%)	5/20 (25%)	2/20 (10%)	15/20 (75%)	3/20 (15%)	0.21 (0.040–1.16)
Karmiris *et al*.^12^	36 weeks clinical 94.5 weeks MRI	24/59 (40%)	21/59 (36%)	14/59 (24%)	1/13 (8%)	—	—	0.12 (0.010–1.00)
Horsthius *et al*. (2011)	—	—	—	—	—	—	—	—
Tozer *et al*.^14^	156 weeks clinical 156 weeks MRI	4/19 (21%)	7/19 (37%)	7/19 (37%)	6/19 (32%)	4/19 (21%)	9/19 (47%)	1/73 (0.40–7.51)
Thomassin *et al*.^15^	160 weeks clinical >104 weeks MRI	26/49 (53%)	10/49 (20%)	13/49 (27%)	16/49 (33%)	—	—	0.43 (0.19–0.96)

CI, confidence interval; MRI, magnetic resonance imaging.

Three papers reported on clinical outcomes in the short term,^7^, ^13^, ^16^ at a median time of 10 weeks (interquartile range [IQR], 8–11 weeks), with a combined sample size of 57 patients. Of 57 patients, 27 (47%) had remission, 4 of 31 (13%) response, and 13 of 31 (42%) no response. Seven papers reported on clinical outcomes in the long term,^8^, ^11^, ^12^, ^14^, ^15^, ^16^, ^17^ at a median time of 52 weeks (IQR, 44–148 weeks), with a combined sample size of 196 patients. Of 196 patients, 88 (45%) had remission, 60 of 180 (33%) response, and 42 of 180 (23%) no response.

### *Radiological outcomes*

Radiological healing was assessed in all nine papers, defined as the absence of high‐signal tracts on T2‐weighted scans (see Tables 2 and 3). Five papers assessed the degree of radiological improvement.^7^, ^8^, ^11^, ^14^, ^17^ This was defined as a decrease in degree of T2 hyperintensity in two papers^7^, ^17^; reduction in number of tracts or cavities in one paper^8^; and as reduction in number of tracts/cavities, reduction in volume of inflammation by ≥10%, **or** decrease in signal intensity in two papers.^11^, ^14^

Three papers reported on radiological outcomes in the short term,^7^, ^12^, ^13^ at a median of 10.5 weeks (IQR, 9–11.25 weeks), with a combined sample size of 60 patients. Of 60 patients, 4 (7%) had healing, 8 of 15 (53%) improvement, and 7 of 15 (47%) no response. Eight papers reported on radiological outcomes in the long term,^7^, ^8^, ^11^, ^12^, ^14^, ^15^, ^16^, ^17^ at a median time of 72 weeks (IQR, 49–84 weeks), with a combined sample size of 148 patients. Of 148 papers, 37 (25%) had healing, 36 of 72 (50%) response, and 18 of 72 (25%) no response.

The van Assche score was assessed in seven papers, with conflicting findings. Some papers reported a statistically significant difference between clinical responders and nonresponders^7^, ^15^, ^17^; others detected a significant difference pre‐ and posttreatment but not between responders and nonresponders.^16^ Others reported no statistically significant different pre‐ and posttreatment^12^, ^13^ or between clinical responders and nonresponders.^11^, ^12^, ^13^

### *Comparison of clinical and radiological outcomes*

The odds ratio of MRI healing *versus* clinical in the short term was 0.10 (95% confidence interval [CI], 0.02–0.39) and in the long term was 0.43 (95% CI, 0.26–0.71). As such, clinical healing is more common than radiological healing, by a factor of 10 in the short term and 2.33 in the long term.

## Discussion

The findings of this review demonstrate the relative infrequency of MRI healing, compared to clinical remission, following biological therapy. The latter was achieved in 47% of patients following biological induction and 45% following a median of 52 weeks' maintenance therapy, consistent with data in existing literature.^5^, ^6^ In contrast, 7% and 25% achieved radiological resolution, and 33% and 50% radiological improvement, in the short and long term, respectively.

Compared to clinical outcomes, the odds of MRI healing were 0.10 (95% CI, 0.02–0.39) at ≤12 weeks, which increased to 0.43 (95% CI, 0.26–0.71) at >12 weeks following anti‐TNFα therapy. The increased frequency of MRI healing and improvement at time points >12 weeks, compared to ≤12 weeks, is consistent with previous studies, which demonstrated a lag in radiological resolution compared to examination findings.^14^

There remains conflicting data regarding the significance of the van Assche score, particularly in real‐world studies, as seen in our review. A universally accepted grading system for pfCD is therefore yet to be developed.

In current practice, clinical remission remains the treatment target. This is despite MRI being the more accurate measure of disease activity, and response to therapy, with previous studies demonstrating persistence of underlying tracts, despite closure of the external opening.^8^ Furthermore, studies have demonstrated the prevalence of loss of response following initial clinical improvement, cited to occur at a median of 40 weeks.^6^

In contrast, Ng *et al*. published data on 26 pfCD patients treated with anti‐TNFα therapy, 5 of whom achieved radiological healing at 6 months.^11^ All five of these patients maintained MRI healing until 18 months. In addition, a study by Tozer *et al*., which included 15 pfCD patients who achieved radiological healing, found that, of the 7 patients who continued biological therapy, 5 maintained MRI healing. In comparison, of the 8 patients who discontinued biologic therapy following healing, 5 had loss of response. This suggests that, while MRI healing may be a relatively less frequent outcome, it is arguably more clinically significant given its prognostic implications for long‐term response to biological therapy. As yet, there have been no studies directly comparing the long‐term clinical outcomes of patients achieving MRI healing to those achieving only clinical remission.

The main limitation for this review is the significant heterogeneity in the nine studies. This included variations in the definitions of the clinical and radiological outcomes assessed, as well as of timing of clinical assessments and follow‐up MRIs. This is likely reflective of the fact that, currently, there is no established protocol for best practice treatment of pfCD. In particular, there were inconsistencies in what constituted clinical and radiological response between the various studies. This made it difficult to analyze data on clinical and MRI response rates, and as such, meta‐analysis was limited to rates of radiological healing compared to clinical remission.

## Conclusion

Despite being a more accurate reflection of underlying disease activity and response to treatment, the radiological outcomes of pfCD remain less well documented. Lack of consensus regarding the definition of clinical and MRI improvement, or a universally accepted radiological grading system, has led to significant variability of end‐points assessed and heterogeneity in reported rates of improvement. Further studies, directly comparing long‐term outcomes following MRI healing to clinical remission, are required to determine whether more aggressive treatment to achieve radiological healing should be commonplace in clinical practice. The findings of this should inform a strict treatment protocol, outlining optimal combined medical and surgical therapy, timing of follow up including repeat MRI scans, and when and how to escalate therapy.
